# Supplementary material for: The Effects and Mechanism of ATM Kinase Inhibitors in Toxoplasma gondii
Source: Int J Mol Sci. 2024 Jun 25;25(13):6947. doi: 10.3390/ijms25136947 (PMC11241798; doi:10.3390/ijms25136947)
Supplement: Supplementary file 1 [file ijms-25-06947-s001.zip › Figures.pdf]

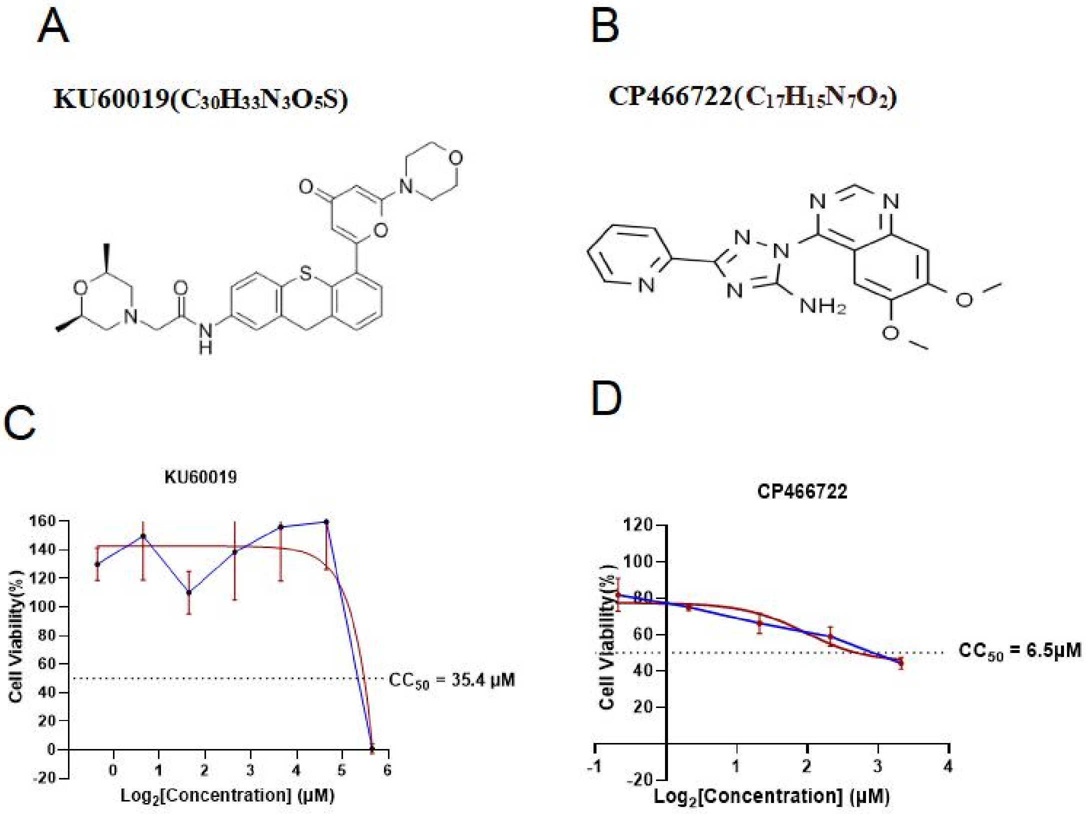

FIGURE S1 Cytotoxicity assay of KU and CP on HFF cells. A-B. The chemical structural formulas of KU and CP. C. The viability of HFF cells treated with different concentrations KU. D. The viability of HFF cells treated with different concentrations CP.

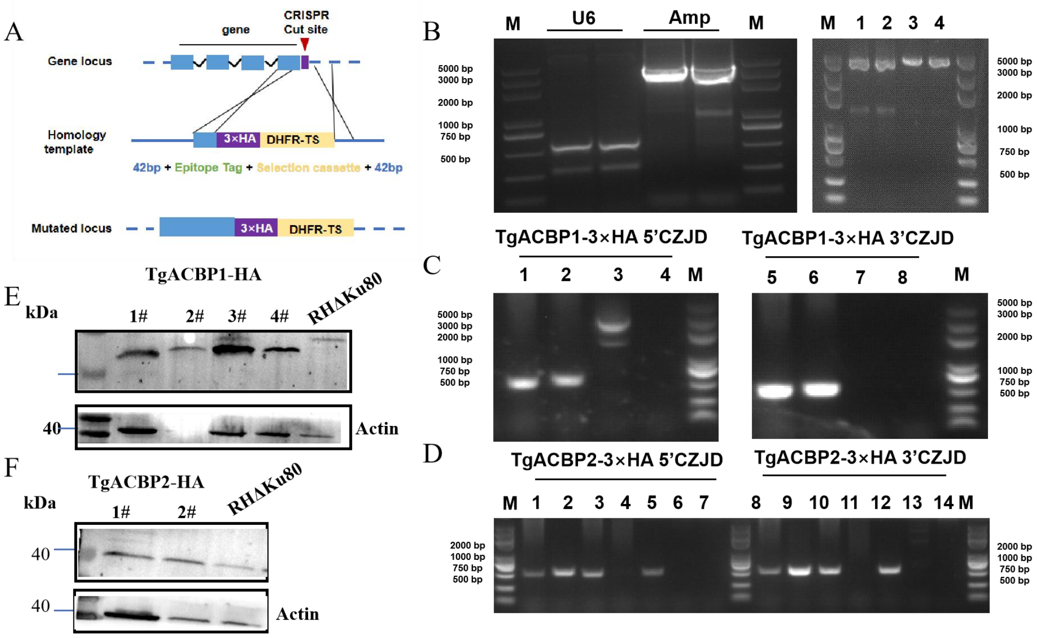

FIGURE S2 Construction of endogenous marked strains. A. Schematic diagram of endogenous marker strains construction. Arrows represent guideRNA specific targeting sites, purple boxes represent 3xHA tags, yellow boxes represent drug screening genes, and the bottom row represents homologous recombination gene sequences. B. The left image shows the TgU6- TgACBP1-gRNA, TgU6-TgACBP2-gRNA, TgACBP1-gRNA-AMP, and TgACBP2-gRNA-AMP fragments in sequence, while the right image shows two TgACBP1-3×HA linearized fragments and two TgACBP2-3×HA linearized fragments each. C. PCR identification of homologous recombination of TgACBP1-HA strain. Lanes 1, 2, 3, and 4 represent the recombined fragments of two TgACBP1-HA monoclonal strains, as well as the 5 'homologous arm recombinations of RHΔ*ku80* and water. Lanes 5, 6, 7, and 8 represent the recombined fragments of two TgACBP1-HA monoclonal strains and the 3' homologous arm recombinations of RHΔ*ku80* and water, respectively. D. PCR identification of homologous recombination of TgACBP2-HA strain. Lanes 1-7 represent the recombined fragments of TgACBP1-HA monoclonal strains, as well as the recombined 5 'homologous arms of RHΔ*ku80* and water, respectively. Lanes 8-14 represent the recombined fragments of TgACBP1-HA monoclonal strains, as well as the recombined 3' homologous arms of RHΔ*ku80* and water, respectively. E. WB identification results of four TgACBP1-HA monoclonal strains. The whole parasite protein of the RHΔ*ku80* maternal strain was used as a negative control, mouse derived HA antibody was used as the primary antibody, sheep anti-mouse IgG was used as the secondary antibody, and Actin protein was used as the sample reference. F. WB identification results of two TgACBP2-HA monoclonal strains.

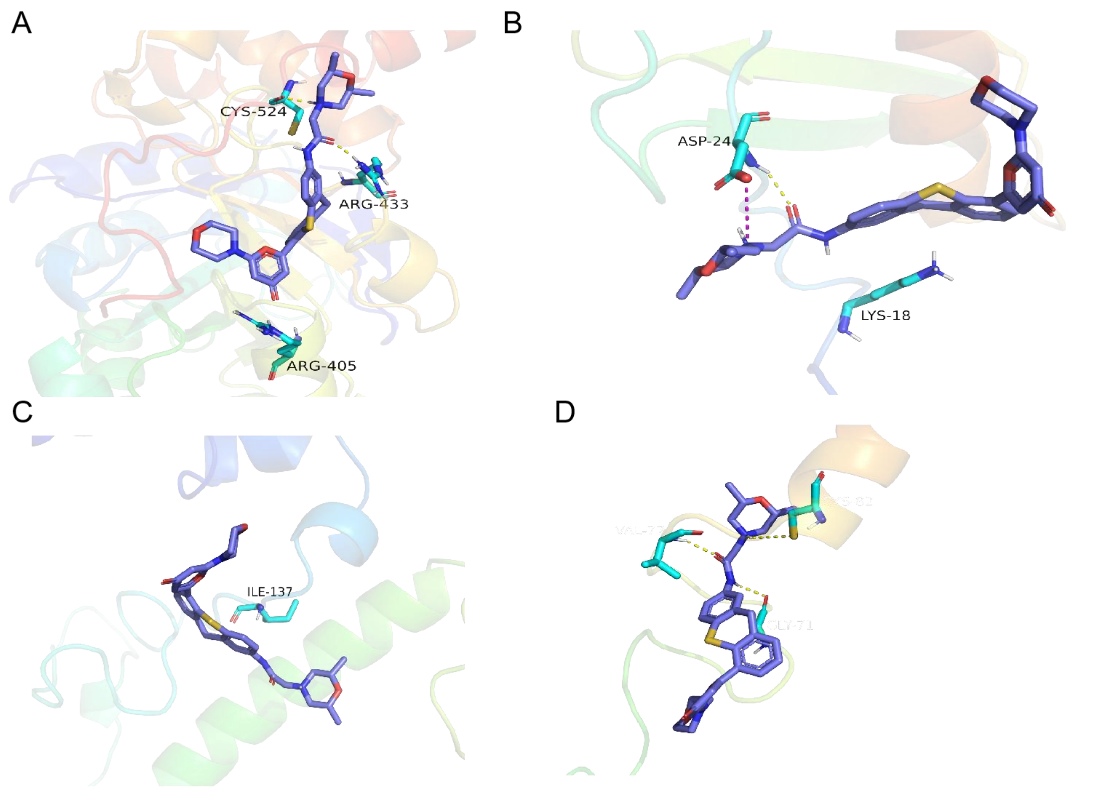

FIGURE S3 Binding patterns of KU and differentially expressed proteins. A-D are the local action views of KU binding to TGGT1_273920、TGGT1_297730、TGGT1_246730 and TGGT1_236920 proteins, respectively. The docking scores are -8, -7.2, -8 and -6.9 kcal/mol. The blue stick represents small molecules, the light blue Cartoon represents binding amino acids, and the yellow dashed line represents hydrogen bonding interactions.

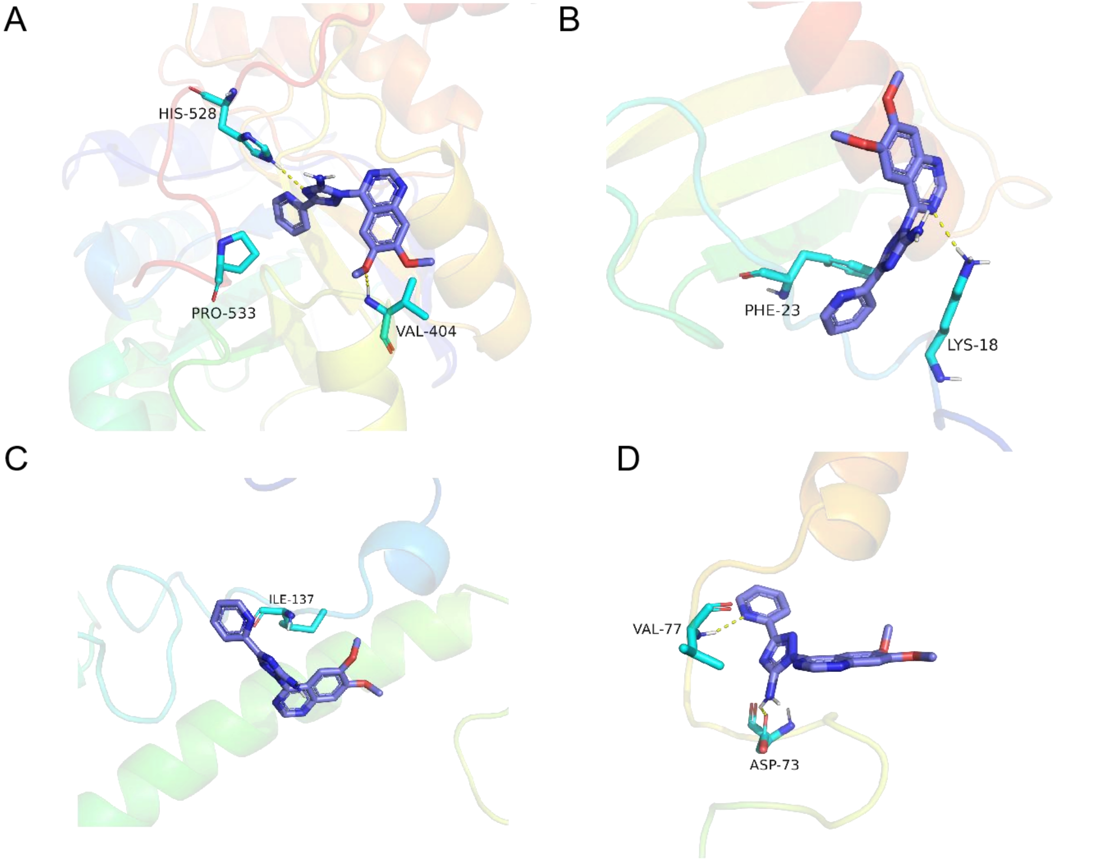

FIGURE S4 Binding patterns of CP and differentially expressed proteins. A-D are the local action views of KU binding to TGGT1_273920 、 TGGT1_297730 、 TGGT1_246730 and TGGT1_236920 proteins, respectively. The docking scores are -7.4, -6.1, -6.4 and -6.2 kcal/mol The blue stick represents small molecules, the light blue Cartoon represents binding amino acids, and the yellow dashed line represents hydrogen bonding interactions.
